# A review of the peripheral proprioceptive apparatus in the larynx

**DOI:** 10.3389/fnana.2023.1114817

**Published:** 2023-02-24

**Authors:** Ignacio Hernández-Morato, Victoria X. Yu, Michael J. Pitman

**Affiliations:** ^1^Department of Otolaryngology—Head and Neck Surgery, Columbia University Irving Medical Center / New York Presbyterian, New York, NY, United States; ^2^The Center for Voice and Swallowing, Department of Otolaryngology—Head and Neck Surgery, Columbia University Irving Medical Center / New York Presbyterian, New York, NY, United States

**Keywords:** laryngeal proprioception, muscle spindles, golgi tendon organs, dysphonia, vocal fold

## Abstract

The larynx is an organ of the upper airway that participates in breathing, glutition, voice production, and airway protection. These complex functions depend on vocal fold (VF) movement, facilitated in turn by the action of the intrinsic laryngeal muscles (ILM). The necessary precise and near-instantaneous modulation of each ILM contraction relies on proprioceptive innervation of the larynx. Dysfunctional laryngeal proprioception likely contributes to disorders such as laryngeal dystonia, dysphagia, vocal fold paresis, and paralysis. While the proprioceptive system in skeletal muscle derived from somites is well described, the proprioceptive circuitry that governs head and neck structures such as VF has not been so well characterized. For over two centuries, researchers have investigated the question of whether canonical proprioceptive organs, muscle spindles, and Golgi tendon organs, exist in the ILM, with variable findings. The present work is a state-of-the-art review of the peripheral component of laryngeal proprioception, including current knowledge of canonical and possible alternative proprioceptive circuitry elements in the larynx.

## Introduction

**Figure 1:**
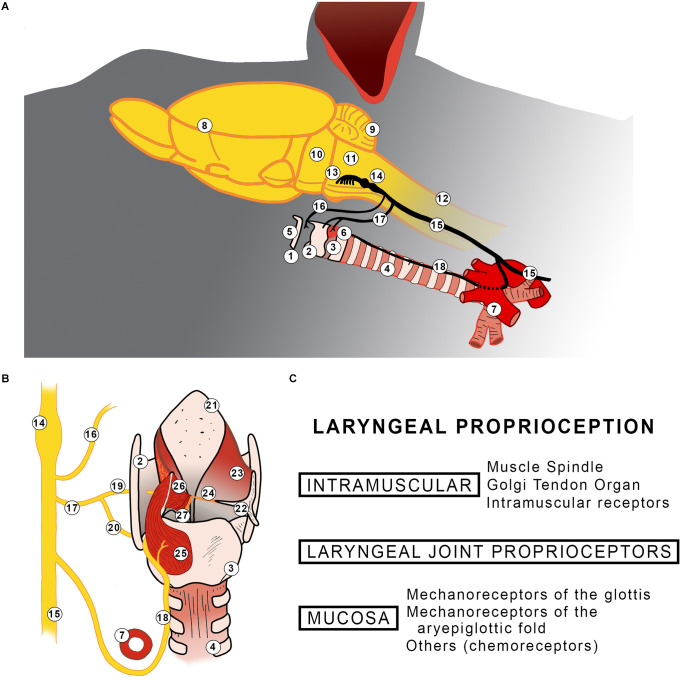
**(A)** Diagram summarizing the location of the larynx and its innervation in rodents **(A)**. A representation of the posterior view of the rat larynx **(B)**. The left side shows innervation, posterior cricoarytenoid, lateral thyroarytenoid, and medial thyroarytenoid muscles. The superior cricoarytenoid and the cricothyroid muscles are not shown. The right side shows aryepiglottic fold having the arytenoid cartilage is displaced and all intrinsic laryngeal muscles excised. Findings of laryngeal proprioceptors reported in the literature **(C)**. 1: Larynx; 2: Thyroid cartilage; 3: Cricoid cartilage; 4: Trachea; 5: Hyoid bone; 6: Cricothyroid muscle; 7: Aorta arch; 8: Telencephalon; 9: Cerebellum; 10: Pons; 11: Medulla oblongata; 12: Spinal Cord; 13: Origin of vagus nerve; 14: Nodose and Jugular ganglion; 15: Vagus nerve; 16: Pharyngeal branch; 17: Superior laryngeal nerve; 18: Recurrent laryngeal nerve; 19: Internal branch of superior laryngeal nerve; 20: External branch of superior laryngeal nerve; 21: Epiglottis; 22: Arytenoid cartilage; 23: Aryepiglottic fold; 24: Vocal fold; 25: Posterior cricoarytenoid muscle; 26: Lateral thyroarytenoid muscle; 27: Medial thyroarytenoid muscle.

The larynx, colloquially known as the voice box, is an organ consisting of several cartilages and muscles that connect the upper respiratory tract (nasal cavity, oral cavity, pharynx) to the lower respiratory tract (trachea, bronchi, lungs; Figure 1). The vocal folds (VF) are structures composed of multiple layers (Table 1 for abbreviations used in the article). The epithelium and superficial lamina propria are together referred to as the mucosa or cover. The intermediate and deep layers of the lamina propria are composed mostly of elastin and collagen, respectively. These layers form the vocal ligament. Beneath the ligament is the thyroarytenoid muscle. The ligament and muscle are referred to as the body of the vocal fold. Anteriorly the vocal ligament inserts into the thyroid cartilage *via* the anterior commissure tendon. Posteriorly the intermediate layer of the lamina propria attaches to the vocal process of the arytenoid *via* a tendon-like structure (Hirano et al., 1987; Hirano, 1993; Paulsen and Tillmann, 1997). The vocal folds abduct and adduct to act as a sphincter to the lower airway. This allows the larynx to participate in physiologic functions such as promoting airflow during respiration, providing airway protection during deglutition, and facilitating phonation (McHanwell, 2008).

**Table 1:** Abbreviations used in this review.

**Abbreviation**	**Definition**
GTO	Golgi Tendon Organs
ILM	Intrinsic Laryngeal Muscles
MuSp	Muscle Spindle
RLN	Recurrent Laryngeal Nerve
SLN	Superior Laryngeal Nerve
TA	Thyroarytenoid Muscle
VF	Vocal Fold

VF motion is controlled by the intrinsic laryngeal muscles (ILM). In humans, these muscles are the thyroarytenoid muscle (TA), lateral cricoarytenoid, posterior cricoarytenoid, interarytenoid, and cricothyroid muscles (Figure 1). Other species demonstrate variations on this. For instance, rodents lack the interarytenoid muscle and instead have the cricoalar and the superior cricoarytenoid muscles (Hernández-Morato et al., 2013a). The ILM contributes to gross movements of the VF, including opening with inhalation and exhalation to allow passage of air between the upper and lower airways, and closing during swallowing to protect the airway from food and saliva. The ILM also contributes to finer-tuned, precise VF movements. This includes micromovements needed to place the VF in the proper position and tension to allow air passing through the larynx to vibrate the vocal folds at specific frequencies. VF position changes constantly during phonation, to facilitate the production of different sounds and pitches (Sasaki, 2006; Song et al., 2009).

The larynx is innervated primarily by two nerves, the superior (SLN) and the recurrent laryngeal nerve (RLN; Figure 1). Both contribute to motor innervation: the external branch of the SLN to the cricothyroid and the RLN to the remaining ILM. These nerves’ contributions to laryngeal sensory innervation appear to vary by species. In humans, the glottic and supraglottic sensation is carried by the internal branch of the SLN while infraglottic sensation is carried by the RLN (McHanwell, 2008). By contrast, in rats, only the SLN carries sensory information (Andrew, 1956; Furusawa et al., 1996; Pascual-Font et al., 2011; Weissbrod et al., 2011; Hernández-Morato et al., 2013a,b).

Adapted to the tasks of the VF, the ILM is among the fastest contracting muscles yet require near instantaneous, constant adjustment (Baken and Orlikoff, 2000; Hoh, 2005; Sasaki, 2006). Such action in turn requires precisely calibrated sensorimotor function. Adjustments of these muscles constantly occur in response to changes in breathing, the sensation of a foreign element such as food, and the need of voice production. These adjustments are influenced by mechano-, chemo-, and baroreceptors of the larynx and airway, as well as aural feedback. While it has been known for some time that somatosensory dysfunction contributes to dysphagia, there is also substantial evidence that disordered laryngeal sensorimotor function plays a role in dysphonia due to VF pathologies, including VF paralysis and spasmodic dysphonia (Sant’Ambrogio et al., 1985; Ludlow et al., 1995; Shiba et al., 1995, 1997; Barkmeier et al., 2000; Jürgens, 2002; Jafari et al., 2003; Ali et al., 2006; Larson et al., 2008; Rosales and Dressler, 2010; Simonyan and Ludlow, 2010, 2012; Hammer and Krueger, 2014; Pitman, 2014; Konczak et al., 2015).

In contrast to the well-studied proprioceptive mechanisms in the body and extremities, proprioception in the larynx is poorly understood despite being studied since the 19th century (Hall, 1833; Head, 1889a,b; Hines, 1927). The following is a literature review detailing the current understanding of the peripheral components of laryngeal proprioception.

## Proprioception

The mechanics of proprioception in skeletal muscles are well-described. Two canonical proprioceptive organs, muscle spindles (MuSp) and Golgi tendon organs (GTO), provide sensory input. MuSp are embedded in the muscle body while GTO are found in muscle tendons. MuSp and GTO collect information about muscle length and tension. This information is relayed to the central nervous system by afferent Group Ia, Ib, and II sensory nerve fibers. Subsequently, the efferent arm is activated with alpha motor neurons activating muscle movement while gamma motor neurons set the gain of the MuSp (Gardner and Johnson, 2014).

Joint capsules and ligaments also contain several sensory organs with afferent nerve terminals that carry sensory information including pain, touch, pressure, and stretch. The main encapsulated mechanoreceptors related to proprioception in these organs are Pacini and Ruffini receptors. While Pacini receptors are activated by compression, Ruffini receptors are stimulated by stretch. In contrast, non-encapsulated free nerve endings provide pain-related sensory input from the joints (Gardner and Johnson, 2014).

## Canonical intramuscular proprioceptors

The presence of canonical intramuscular proprioceptors (MuSp and GTOs) in the ILM is unclear. In particular, despite exploration since the 1960s, evidence of laryngeal MuSp remains controversial (Table 2).

**Table 2:** Summary of the proprioceptive findings in the larynx.

**Anatomical finding**	**Species**	**Experimental method**	**Where in the larynx?**	**Reported finding**	**Cited literature**
Muscle spindles	human	Histological staining	ILM	Several muscles	Hines (1927), Keene (1961), Rossi and Cortesina (1965), and Baken (1971)
		Staining for electronic microsopy analysis	ILM	IA	Katto et al. (1987)
		Histological staining	ILM	TA	Konig and von Leden (1961) and Sanders et al. (1998)
		Staining intrafusal fibers for identification of muscle spindles	ILM	IA	Tellis et al. (2004)
	guinea pig	Staining for electronic microsopy analysis	ILM	PCA	Desaki et al. (1997) and Desaki et al. (1998)
	marmoset	Staining for electronic microsopy analysis	ILM	PCA	Desaki et al. (1997)
	human	Histological staining for myosin heavy chains of the muscle fibers	ILM	Presence of tonic muscle fibers but no muscle spindles found in the TA	Brandon et al. (2003a)
Mechanoreceptors inside ILM	cat	Electrophysiology recorded from the ILM	ILM	Positive recording from several ILM	Bianconi and Molinari (1962), Sampson and Eyzaguirre (1964), Abo-El-Enein and Wyke (1966), Kirchner and Suzuki (1968), Storey (1968), and Shiba et al. (1997)
	dog	Electrophisiology recorded from the ILM	ILM	Positive recording from TA	Mårtensson (1964)
Receptors at the mucosa	human	Staining for electronic microscopy analysis	Epithelia of the laryngeal mucosa	Encapsulated nerve structures under the epithelia at the vocal folds	Nagai (1987) and Nagai (1989)
	cat	Histological staining	Epithelia of the laryngeal mucosa	Nerve endings at the mucosa	Adzaku and Wyke (1979)
	cat	Electrophysiology recording from the laryngeal nerves	Epithelia of the laryngeal mucosa	Presence of mechanoreceptors in the laryngeal mucosa	Sampson and Eyzaguirre (1964), Kirchner and Suzuki (1968), Suzuki and Kirchner (1969), Davis and Nail (1987), and Andreatta et al. (2002)
	dog	Electrophysiology from the laryngeal nerves	Epithelia of the laryngeal mucosa	Presence of mechanoreceptors in the laryngeal mucosa	Mårtensson (1964), Sant’Ambrogio et al. (1983, 1991), and Mathew et al. (1984)
Receptors at the epiglottis	rat	Electrophisiology from the laryngeal nerves	Epiglottis	Presence of receptors at the epiglottis	Andrew (1954) and Storey (1968)
	rat	Anatomical and histological staining analysis of the nerve fibers	Epiglottis	Presence of receptors at the epiglottis	Andrew and Oliver (1951)
Mechanoreceptors at the laryngeal joints	cat	Electrophysiology and histological staining	Joints of the laryngeal cartilagues	Presence of mechanoreceptors at the laryngeal joints	Kirchner and Wyke (1965) and Kirchner and Suzuki (1968)

ILM, intrinsic laryngeal muscles; IA, interarytenoid muscle; TA, thyroarytenoid muscle; PCA, posterior cricoarytenoid muscle.

### Electrophysiological studies

In the 1960s electrophysiological studies posited the presence of mechanoreceptors within the ILM. Most of these studies were performed in cats where investigators recorded afferent discharges of the SLN and RLN in the setting of various stimuli to the VF and surrounding laryngeal structures (Bianconi and Molinari, 1962; Mårtensson, 1964; Sampson and Eyzaguirre, 1964; Abo-El-Enein and Wyke, 1966; Kirchner and Suzuki, 1968; Storey, 1968). Stimuli included both natural stimulation, such as respiration, and experimentally provoked stimulation, including direct nerve stimulation and artificial stretching and probing of the ILM (Table 2). Further, investigators used various methods to determine whether the mechanoreceptive sensory input arose from the mucosa, joint, or muscle. For instance, to eliminate mucosal mechanoreceptive input from the signal, Kirchner and Suzuki (1968) anesthetized the mucosa of the cat larynx. To eliminate input from the joints, Abo-El-Enein disarticulated the joints prior to the recording (Abo-El-Enein and Wyke, 1966).

Such electrophysiological evidence is indirect evidence of intramuscular proprioceptors, and the authors of these studies offer different degrees of conviction in their conclusions about the presence of mechanoreceptors in the ILM. The most specific conclusions are those of Abo-El-Enein and Wyke (1966) in their delineation of the “laryngeal myotactic reflex.” The authors found, through electromyography of the ILM and extrinsic laryngeal muscles, that stretch applied to one ILM results in sustained motor unit activity changes not only in the stretched muscle, but also in other muscles—a myotactic reflex. This reflex could be facilitatory or inhibitory, depending on the degree of stretch. Further, they speculated facilitatory mechanoreceptors are in parallel with muscle fibers while inhibitory ones are in series (Abo-El-Enein and Wyke, 1966). These conclusions were not made in conjunction with morphologic studies. Other authors of electrophysiological studies state that while intramuscular receptors are a possibility, stretch responses are likely also mediated by deep mucosal or submucosal receptors or connective tissue receptors (Hirose, 1961; Mårtensson, 1964; Suzuki and Kirchner, 1969; Table 2).

### Visualizing intramuscular proprioceptors

Investigators have also performed studies using traditional stains (e.g., hematoxylin and eosin) and immunohistochemistry of the ILM to visualize the proprioceptive organs. These studies have been performed across multiple species including human, dog, nonhuman primates, and guinea pig. These studies have specifically focused on identifying MuSp and demonstrated interspecies and intraspecies variation regarding whether MuSp exist in the ILM. For instance, investigators have reported histological evidence of MuSp in the ILM of humans, guinea pigs, and marmosets, but not in the ILM of dogs (Keene, 1961; Konig and von Leden, 1961; Mårtensson, 1964; Rossi and Cortesina, 1965; Katto et al., 1987; Desaki et al., 1997; Sanders et al., 1998; Desaki and Nishida, 2006).

There is particular controversy over MuSp in the human ILM. Specifically, studies have yielded conflicting findings regarding whether MuSp exist and in what distribution. The most studied of the ILM is the TA. The history of this investigation through 1998 is expertly described by Sanders et al (Sanders et al., 1998). In summary, between 1950 and 1987, multiple groups observed MuSp in the TA using various traditional stains including hematoxylin and eosin, silver, and trichrome stains (Keene, 1961; Konig and von Leden, 1961; Rossi and Cortesina, 1965; Baken, 1971; Katto et al., 1987; Baken and Orlikoff, 2000). Others have found MuSp to be absent in the TA (Fernand and Young, 1951; Murray, 1957; Table 2).

Sanders et al. advanced this body of work in 1998 with a hematoxylin and eosin-based investigation demonstrating that MuSp not only exist in the TA but are specifically concentrated in the superior medial quadrant of the TA. This is consistent with previous findings that MuSp in the TA are located in close proximity to the vocal ligament (Konig and von Leden, 1961; Rossi and Cortesina, 1965). Further, they note this quadrant contains one of the highest MuSp -to-muscle mass ratios in the body. The group proposes that the composition of this “compartment” of the TA—a combination of MuSp and slow muscle fibers—facilitates posture and fine-tuned movement of the VF.

Most recently in 2003, Brandon et al. (2003a) provide a countering view using myosin heavy chain immunohistochemistry analysis of the TA. The group’s work is based on the premise that intrafusal fibers of MuSp are formed of two isoforms of myosin heavy chain—neonatal and tonic. These isoforms are not typically found in postnatal extrafusal fibers. The authors found that the TA contained only small-diameter, isolated tonic fibers, but none in the form of MuSp, in contrast to control infrahyoid muscle which contained clear neonatal- and tonic-positive MuSp. The other ILM are less studied. Authors agree that MuSp exist in the interarytenoid muscle using both traditional stains and myosin heavy chain immunohistochemistry (Katto et al., 1987; Tellis et al., 2004) Findings for the posterior cricoarytenoid and lateral cricoarytenoid muscles are mixed (Keene, 1961; Rossi and Cortesina, 1965; Katto et al., 1987; Brandon et al., 2003a, b).

The authors provide possible explanations for the variable findings. Just as unique extrafusal muscle fiber isoforms have been described in cranial muscles including the masseter, extraocular, and laryngeal muscles, groups have observed that the MuSp of ILM may be structurally different from stereotypical MuSp and thus may be more difficult to identify (DelGaudio et al., 1995). Katto et al. describe a thinner capsule and unique chain fiber features (Katto et al., 1987). Sander et al. observe that MuSp in the TA are shorter and contain fewer intrafusal fibers than skeletal muscle MuSp (Sanders et al., 1998). Tellis et al. found that interarytenoid muscle MuSp do not always contain bag 2 fibers, sometimes have extrafusal fibers coursing through part of the MuSp, and sometimes form branching complexes as is seen in other cranial muscles like the masseter (Tellis et al., 2004). Tellis et al. further elaborate that the lack of bag 2 fibers implies that interarytenoid muscle MuSp facilitate rapid contraction of the muscle, as opposed to postural maintenance. Authors studying ILM MuSp of other species have also noted they differ from skeletal muscle MuSp (Desaki et al., 1997, 1998; Desaki and Nishida, 2006). Meanwhile, Brandon et al. (2003a) who conclude the TA does not contain MuSp by myosin heavy chain immunohistochemistry, note the region of the TA adjacent to the vocal ligament, where previous authors have described the presence of MuSp, contains high connective tissue content. This connective tissue surrounds intermingled extrafusal fibers to create the appearance of MuSp. However, these structures neither stain for neonatal or tonic myosin heavy chain, nor have true capsules and thus are not traditional MuSp. Another possibility is that MuSp are only rarely expressed in ILM other than the interarytenoid muscle.

Overall, GTO have been rarely explored in the ILM, with one group observing encapsulated GTO-like nerve structures on electron microscopy (Nagai, 1987, 1989).

## Alternative intramuscular proprioceptors

As an alternative to MuSp and GTO, could the ILM have a unique intramuscular proprioceptive apparatus altogether? Brandon et al. (2003a) speculate the TA may contain “spindle-like” mechanoreceptors which convey proprioceptive information and that perhaps modified extrafusal fibers serve proprioceptive purposes. The intrafusal fibers that comprise MuSp are, after all, specialized muscle fibers (Gardner and Johnson, 2014). Additionally, Rossi describes a unique entity consisting of spiral nerve endings around single muscle fibers in the human ILM (Rossi and Cortesina, 1965).

There are other muscles innervated by cranial nerves which also appear to lack canonical proprioceptive organs and in which alternatives have been proposed:

(a) Extraocular muscles: MuSp are present in the extraocular muscles of some mammals (e.g., humans and sheep) yet absent in others (e.g., some species of monkeys and apes, dogs, cats, and rabbits; Cooper and Daniel, 1949; Lukas et al., 1994; Ruskell, 1999; Blumer et al., 2006; Lienbacher and Horn, 2012) Human extraocular MuSp, though present, are noted to be simplistic in structure (Lienbacher and Horn, 2012). In species without MuSp, terminal nerve ending formations called palisade endings have been proposed as alternative proprioceptors (Billig et al., 1997; Eberhorn et al., 2005; Büttner-Ennever et al., 2006; Lienbacher and Horn, 2012). First described in cats and monkeys in the 1900s, palisade endings are myelinated nerve fibers that enter the muscle centrally, run to the distal tip of the muscle, branch, and then end in the myotendinous junction, surrounding this area in a palisading pattern and terminating on myotendinous cylinders, loose connective tissue caps encapsulating single muscle fiber-tendon junctions (Ruskell, 1999; Lukas et al., 2000; Konakci et al., 2005). However, studies debate whether palisade endings are truly proprioceptive or motor in function. Specifically, studies have shown that some palisade endings nerve terminals contain choline acetyltransferase vesicles and degenerate after injury to extraocular motor nuclei, suggesting an effector role (Lukas et al., 1994; Eberhorn et al., 2005; Büttner-Ennever et al., 2006; Lienbacher and Horn, 2012).
(b) Facial mimetic muscles and the superior pharyngeal constrictor: MuSp have been identified in neither the facial mimetic muscles nor the superior pharyngeal constrictor (Stål et al., 1987, 1990; Kuehn et al., 1990; Happak et al., 1994; Goodmurphy and Ovalle, 1999; de Carlos et al., 2013; Cobo et al., 2017; May and Bramke, 2021). In the face, a myocutaneous mechanoreceptive component to proprioception has been proposed (Connor and Abbs, 1998; May and Bramke, 2021). May and Bramke describe the insertion of muscle fibrils of the orbicularis oris inserting directly into collagen networks of the reticular dermis or hypodermis-reticular dermis junction, suggesting a possible mechanism for the transduction of changes in skin tension, though these insertions lack superstructures noted of canonical mechanosensory organs (May and Bramke, 2021). Additionally, the Vega group has described intramuscular corpuscular structures that stain positive for putative mechanoproteins ASIC2 and TRPV4 in the facial mimetic and superior pharyngeal constrictor muscles, which may represent a novel intramuscular proprioceptor specific to these muscles (de Carlos et al., 2013; Cobo et al., 2017).

## Joint proprioceptors

The human larynx contains three paired encapsulated joints: the cricothyroid, cricoarytenoid, and arytenocorniculate joints (McHanwell, 2008). Additionally, the thyroepiglottic ligament joins the epiglottis to the thyroid cartilage. Given these joints’ role in the articular reflexes of the larynx, studies from the 1950s and 60s have investigated the presence of proprioceptors in the laryngeal joints using electrophysiological and histochemical techniques. They demonstrated that laryngeal joints and ligaments contain Pacini and Ruffini organs, and numerous free nerve endings (Andrew, 1954, 1956; Kirchner and Wyke, 1965; McHanwell, 2008; Table 2). Further, in contrast to other joints in the body, Pacinian organs are more abundant than Ruffini organs in the encapsulated laryngeal joints (Kirchner and Wyke, 1965).

Studies investigating the source of sensory innervation to the laryngeal joints show the encapsulated joints receive input from the internal branch of the SLN, and the cricothyroid joint may also receive input from the RLN (Kirchner and Wyke, 1965; Storey, 1968). The thyroepiglottic ligament is also innervated by the internal branch of the SLN (Andrew and Oliver, 1951; Andrew, 1954, 1956).

## Mucosal mechanoreceptors

Many afferent receptors are present in the laryngeal mucosa (Adzaku and Wyke, 1979; Bradley, 2000; Foote and Thibeault, 2021). Electrophysiological studies and staining with mechanoreceptor marker Piezo2 demonstrate that a subset of these receptors is mechanosensitive (Sampson and Eyzaguirre, 1964; Storey, 1968; Davis and Nail, 1987; Widdicombe, 2001; Prescott et al., 2020). While these mucosal mechanoreceptors have not been directly implicated in proprioception, their anatomic distribution and contribution to laryngeal reflexes suggest a potential role in the afferent arm of the laryngeal proprioceptive arc (Table 2).

Mechanoreceptors are not uniformly distributed throughout the laryngeal mucosa and are focused in and around the vibratory and mobile elements of the larynx. Electrophysiological studies in cats and rabbits demonstrate that mechanoreceptors are concentrated in the laryngeal mucosa in the glottal area above and below the vocal folds. Some are sensitive to vibration from the VF and others to changes in pressure from airflow (Sampson and Eyzaguirre, 1964; Storey, 1968; Sant’Ambrogio et al., 1983, 1991; Mathew et al., 1984; Davis and Nail, 1987; Mortola and Piazza, 1987; Shiba et al., 1997; Bradley, 2000; Widdicombe, 2001). Additionally, Piezo2 staining of rodent larynges shows that the aryepiglottic fold and the vocal folds contain clusters of mechanoreceptors (Prescott et al., 2020). Further, conglomerates of VGLUT-positive staining are observed in the mucosa overlying and laryngeal surface of the epiglottis of rats (Soda and Yamamoto, 2012; Takahashi et al., 2016).

Authors also find evidence these mucosal receptors play a role in the laryngeal adductor response. Specifically, Andreatta et al. show that mucosal mechanoreceptors play an important role in the laryngeal adductor response, which is diminished when the larynx is stripped of mucosa (Mårtensson, 1964; Andreatta et al., 2002).

## Conclusion

The study of laryngeal proprioception is fragmented in method and species. Nevertheless, a few unifying conclusions can be drawn from the current knowledge. First, canonical proprioceptive organs are rarely expressed in the ILM, aside from the interarytenoid muscle. Second, this is consistent with the finding that other cranial muscles, such as extraocular and facial mimetic muscles, lack MuSp but demonstrate evidence of non-canonical proprioceptive organs. Third, given the lack of canonical elements in ILM, an alternative proprioceptive mechanism may govern the coordination of laryngeal movement, and this requires further study.

## Author contributions

IH-M and VY: conceptualization, formal analysis, and writing—original draft. IH-M, VY, and MP: writing—review and editing. IH-M: visualization. MP: supervision, project administration, and funding acquisition. All authors contributed to the article and approved the submitted version.
