# Corrigendum: A review of the peripheral proprioceptive apparatus in the larynx

**DOI:** 10.3389/fnana.2023.1206526

**Published:** 2023-05-12

**Authors:** Ignacio Hernández-Morato, Victoria X. Yu, Michael J. Pitman

**Affiliations:** ^1^Department of Otolaryngology – Head and Neck Surgery, Columbia University Irving Medical Center / New York Presbyterian, New York, NY, United States; ^2^The Center for Voice and Swallowing, Department of Otolaryngology - Head & Neck Surgery, Columbia University Irving Medical Center / New York Presbyterian, New York, NY, United States

**Keywords:** laryngeal proprioception, muscle spindles, golgi tendon organs, dysphonia, vocal fold

In the published article, there was an error regarding the affiliations for Michael J. Pitman. As well as having affiliation(s) 1 Department of Otolaryngology – Head and Neck Surgery, Columbia University Irving Medical Center / New York Presbyterian, New York, NY, United States., they should also have “The Center for Voice and Swallowing, Department of Otolaryngology - Head & Neck Surgery, Columbia University Irving Medical Center / New York Presbyterian. New York, NY, United States.”

In the published article, there was an error in the Funding statement. The Funding Statement was absent. The correct Funding statement appears below.

This work was supported by the National Institutes of Health: 1R01DC018060.

In the published article, there was an error. In Table 2 some species names in the second column are in capital letters.

A correction has been made to Table 2, second *column*, second row. This field previously stated:

“Human”, the correct name is “human”.

In Table 2, second *column*, sixth row. This field previously stated:

“Guinea Pig”, the correct name is “guinea pig”.

In Table 2, second *column*, seventh row. This field previously stated:

“Marmoset”, the correct name is “marmoset”.

In Table 2, second *column*, eleventh row. This field previously stated:

“Human”, the correct name is “human”.

The authors apologize for this error and state that this does not change the scientific conclusions of the article in any way. The original article has been updated.

